# Nutritional Considerations for Para-Cycling Athletes: A Narrative Review

**DOI:** 10.3390/sports9110154

**Published:** 2021-11-16

**Authors:** Joelle Leonie Flueck

**Affiliations:** Institute for Sports Medicine, Swiss Paraplegic Centre, CH-6207 Nottwil, Switzerland; joelle.flueck@paraplegie.ch; Tel.: +41-41-9396617

**Keywords:** paralympic, cycling, sports nutrition, disability, supplementation

## Abstract

Para-cycling is a sport including athletes with different disabilities competing on the track and on the roads using bicycles, tandems, tricycles, and handbikes. Scientific literature in this special population is scarce, especially in the field of sports nutrition. This review summarizes the physiological aspects and demands of para-cycling. This information together with the existing literature on nutritional interventions in this population, helps to discuss the nutritional considerations. To date, only a limited amount of recommendations are available for this population. In most para-cycling athletes, a reduction in active muscle mass and consequently a reduction in resting energy expenditure occurs, except for visually impaired athletes. Furthermore, carbohydrate and protein intake and hydration, supplementation, heat, and weight loss need to be tailored to the disability-specific adaptations such as the reduced active muscle mass, neurogenic bladder, and bowel, a reduced metabolic cost during exercise, and a higher risk of micronutrient deficiency.

## 1. Introduction

Compared to the Olympics, Paralympic athletes represent a minority under all professional athletes. However, Paralympic sports development was huge in the past few years, with an increasing number of athletes (e.g., Winter Games: 53 athletes in 1976 to 569 athletes in 2018) competing at the Paralympic Games [1]. The athletes’ performance level and training volume developed impressively over the last decades [2]. Research on the material (e.g., prosthesis, carbon racing wheelchair, etc.) emerged, and World records got broken in the process [3,4]. However, research on sports nutrition in para-athletes is lacking, and therefore, evidence-based nutritional considerations are not available [5]. In contrast, health, training, and nutrition are affected by the unique physiological aspects of athletes with disabilities such as visual impairments, amputation, spinal cord injuries (SCI), cerebral palsy (CP) as well as other disabilities [6,7,8]. For example, athletes with SCI show reduced energy expenditure during rest and exercise due to a lower amount of active muscle mass [9]. Physiological mechanisms behind training adaptations on a muscular, cardiovascular or metabolic level might be the same as in able-bodied athletes. Therefore, fueling strategies need to be tailored to the individual demands of the sport itself and the specific disability type of the athlete. Para-cycling is a sport that includes several disabilities classifying them into different categories (Table 1). This review aims to summarize the physiological consequences of the different disabilities and relate them to nutritional considerations. This should help to identify future research questions and provide some practical considerations when working with para-cycling athletes. 

## 2. Disability-Specific Physiological Aspects in Para-Cycling Athletes

### 2.1. Spinal Cord Injury 

The incidence of a SCI leads to an involuntary loss of sensory, motor, and autonomic function [12]. The loss of motor innervation results in a loss of active muscle mass, increased fat mass, and decreased bone mineral density in the affected extremities [13]. The physiological aspects and demands of different wheelchair sports lead to distinct adaptations and bring about different body composition types [14]. Handcycling athletes showed a substantially lower fat-free mass (46.1 ± 8.3 kg) [14] compared to professional cyclists (70.9 kg fat-free mass) [15]. The reduced active muscle mass affects the metabolic rate by decreasing resting energy expenditure (REE) [16]. Abel, Kroner, Rojas Vega, Peters, Klose and Platen [17] measured a REE of 1521.6 ± 294.0 kcal/d in handcycling athletes, whereas male able-bodied well-trained cyclists showed a REE of 1768 kcal/d [18]. This reduction in REE and metabolic rate during daily activities diminishes total energy expenditure (TEE) [9]. Counteracting this reduction with a lower dietary intake often leads to macro-and micronutrient deficiencies in elite athletes with SCI [19,20,21]. Moreover, a neurogenic bladder and bowel, gastrointestinal (GI) dysbiosis, and a longer GI transit time further affect nutritional composition and energy intake [22,23]. Secondary complications such as urinary tract infections or pressure ulcers might lead to a loss of training days [24] and impair performance development. 

### 2.2. Cerebral Palsy

This disability involves a movement dysfunction attributed to damage in the motor cortex, cerebellum, or basal ganglia. Athletes often show spasticity and reduced ability to stretch the extremities, ataxia, tremor, or an inability to control the movement. Further medical issues included pain, fatigue (i.e., inability to sustain power), degenerative arthritis, vision and hearing impairments, or oral malformation. In thirty individuals (non-athletes) with CP, total energy expenditure was measured using the doubly labeled water method as well as REE and dual-x-ray absorptiometry (DXA) to determine TEE [25]. The TEE was highly variable due to the ambulation status of the participants. Therefore, factors such as sex, ambulation status, body composition (i.e., fat-free mass), and other medical conditions need some consideration in CP. Furthermore, it is described that children with CP are at a high risk for under- or malnutrition, which has some further effect on bone health, growth, physical and cognitive development [26,27]. Nutritional assessment, including oral-motor function, body composition, and energy balance, seems to be crucial in children and athletes with CP [27]. 

### 2.3. Amputees

An amputation (e.g., lower-leg) reduces body mass, body surface area, and active muscle mass dependent on the amputation. Scaramella, Kirihennedige Scaramella, Kirihennedige and Broad [5] assume a reduction of REE in lower leg amputees due to the reduction in fat-free mass. No data is available for athletes with an amputation to confirm this hypothesis. Mengelkoch, Kahle and Highsmith [28] reported a higher energy cost in above-knee amputees compared to non-amputees during walking. No study on energy expenditure during cycling in athletes with amputation has been found. But it seems that there is a different gross efficiency in cycling with an amputation or neurological impairment compared to able-bodied cyclists [29]. This changes not only pacing but also leads to considerably different performance time between C1 to C3 compared to able-bodied athletes in 1 km time trial performance [29]. Thus, even with a higher energy cost during walking, power output during cycling might be lower, resulting in reduced daily energy expenditure. 

Regarding bone health, a lower ground reaction force reduces bone load and results in a decreased bone mineral density [5]. In addition, there seems to be a higher risk for pressure sores, discomfort, and infection at the contact site of the prosthesis [6]. Particularly, this needs attention regarding training volume, fitting of the prostheses, and nutrition and infect prophylaxis. 

### 2.4. Visual Impairments

The most common causes for visual impairments are degenerative eye disease, glaucoma, diabetic retinopathy, or cataract [6]. Visually impaired athletes show similar physiological conditions compared to their able-bodied counterparts, and it is not expected that REE is substantially reduced [6]. However, they often show a reduced physical performance and lower balance, and a lower VO_2max_ in relation to the extent of the disability [30]. 

## 3. Physiological Demands of Para-Cycling

Disciplines of para-cycling include track and road racing similar to the Olympic cycling program (Table 1). Several disciplines are limited to some categories, as handcycling athletes are not allowed to compete on the track. Moreover, the differences between upper body (i.e., handcycling) and lower body exercise (i.e., cycling) result in a different exercise energy expenditure even though similar levels of glycogen depletion were reported in wheelchair athletes compared to able-bodied individuals [31]. In general, a 40 to 70% reduction in metabolic costs was found in various wheelchair sports compared to their able-bodied counterparts [9]. A summary of the exercise energy expenditure during handcycling and arm cranking is provided in Table 2. Most of these studies were performed with non-elite athletes, and therefore, a slightly higher metabolic cost can be anticipated in elite handcyclists. Athletes with minimal disabilities, such as visually impaired athletes, are expected to produce a similar metabolic cost for the same workload as their able-bodied counterparts. Professional able-bodied cyclists showed an energy cost of 11.5 to 14.9 MJ during preseason training sessions with a total energy expenditure of around 20 MJ/d [32]. Whereas daily energy expenditure of 32.3 ± 3.2 MJ/d was shown during a stage race [15]. Any para-cyclists will not reach those values due to shorter race duration and lower metabolic costs. Still, it shows that training or racing in para-cycling might result in high energy expenditure, especially during the preseason with a high training volume or during stage races. 

A race analysis in para-cyclists (i.e., T2, C4, C5, and B (see Table 1)) revealed a mean time to complete 20 km of 36.7 min (power output: 199 ± 42 W) [33] whereas non-elite able-bodied cyclists completed the same distance in 30 min (mean power: 325 W) [34]. A recent review [35] summarized the time to complete the time trial at the Rio Paralympic Games in different handcycling categories for male and female athletes separately. Duration ranged between 27 and 31 min in the time trial and between 75 and 98 min in the road race. Even though race duration seems shorter due to a lower amount of active muscle mass, glycogen storage capacity seems to be lower [23]. Furthermore, athletes seem to rely more on CHO oxidation (Table 2) which shows the need for CHO fueling before and during a race [23]. 

In track racing, the data analyzed during several different World Championships and the 2016 Paralympic Games in Rio showed a duration of 70 s for 1 km [40]. Another study investigating the splits of C1 to C3 categories in Paralympic track cycling resulted in duration between 73 s (i.e., C3) and 82 s (C1) [29]. Corbett [41] showed a mean time of 63 s in elite able-bodied athletes competing in several World Championship races. These findings suggest that all athletes, whether competing in para-cycling or in Olympic track cycling, rely mostly on glycolytic energy production even though power output and speed are markedly different. 

## 4. Nutritional Considerations for Para-Cycling Performance

The summarized data on disability-specific considerations and energy expenditure during exercise shows why an individual nutritional assessment is required for each athlete in order to give any nutrition-related recommendations. For each athlete, the disability and its consequence on GI functioning, metabolism, body composition as well as other secondary medical issues need to be assessed in a first step. One should also consider including the assessment of REE by indirect calorimetry and body composition (e.g., DXA-scan or sum of skinfolds) [6,16]. Information on muscle functioning (e.g., lesion level in SCI or medical diagnosis in other athletes with a disability) as an estimate for active muscle mass and glycogen storage capacity or ergospirometric data from performance diagnostics (where available) further help to estimate energy expenditure and fueling requirements during training and racing [23]. A nutrition plan for a para-cycling athlete with SCI (Table 3) exemplifies how to distribute energy intake regarding to training volume and content in an athlete with a reduced energy requirement (i.e., REE about 1450 kcal/d) but with the goal to maximize training adaptation [5]. 

Athletes with SCI might need some further attention concerning GI comfort, bowel management, appetite and satiety. Due to neurogenic bowel, an optimized GI management seems to be of interest in the athletes with GI disturbances (e.g., athletes with SCI) to minimize GI issues [22]. In addition, nutrition-related hormones might be altered as leptin levels were 32% higher in individuals with SCI compared to able-bodied individuals [42]. This affects satiety and eating patterns. In the past, it has been shown that the nutritional intake in Paralympic track and field athletes (e.g., amputation, CP and visual impairment) was poor regarding fruit and vegetables consumption [43]. In addition, in professional able-bodied cyclists, a 30% lower energy intake was observed compared to energy requirements during a 6-day preseason training camp [32]. In this study, carbohydrate intake was extremely low (13 g/h) during long endurance training sessions (e.g., 150 km) which exemplifies the poor adherence of athletes to sports nutrition guidelines. In further research, sports nutrition knowledge has been evaluated in able-bodied and wheelchair athletes and showed a lack of knowledge and understanding for the basic principles of nutrition in sports and exercise [44]. Consequently, the adaptation and education of general healthy nutritional practices and the development of sports nutrition knowledge is a priority when working with para-cycling athletes. 

### 4.1. Micronutrients

Key micronutrients to consider in para-cycling athletes are the same as for able-bodied athletes. Iron is needed for oxygen transport, and therefore, an iron deficiency would diminish endurance performance [5,45]. As para-athletes per se are at a higher risk for low bone mineral density due to less weight-bearing activities and low impact forces [5], vitamin D and calcium intake are even more important compared to able-bodied athletes [46]. Furthermore, in elite wheelchair athletes from various disciplines, vitamin D deficiency occurred very often mainly because of skin protection through clothing and staying indoor due to impaired thermoregulatory function [20]. Micronutrient intake in Paralympic athletes [47,48] was lacking, especially nutrients such as iron, vitamin D, calcium, and potassium, and might be linked to low fruit and vegetable consumption [43] as well as low energy consumption in general [21]. To summarize, the intake and status of micronutrients should be considered with respect to physical performance and general health regardless of the disability. 

### 4.2. Carbohydrate Intake as a Fuel for Performance

As a source for energy production, carbohydrate (CHO) storage and oxidation are extremely important before, during, and after training sessions (Table 4). Especially for high-intensity exercise performance, the glycolytic pathway through glycogen storage remains the primary energy source [45]. Nevertheless, the daily variation of the CHO intake based on training volume and intensity might be important to ensure optimal performance and body composition [49]. Those current guidelines (Table 4) need some adaptations to the demands of para-athletes. Glycogen stores in athletes with SCI, CP, or amputees might be smaller due to lower active muscle mass. Therefore, it seems reasonable that CHO guidelines for these athletes are tailored based on their active muscle mass, energy requirements, and fat-free mass. There is a need to differentiate between the energy/CHO needs of handcycling (e.g., upper body exercise) and other para-cycling athletes (e.g., lower body exercise). The amount of glycogen stored as well as the energy cost during the activity might differ. Furthermore, if gastric emptying [50] is reduced and GI transit time prolonged [51] in athletes with SCI, CHO guidelines for fueling during the day as well as during training or competition might change. Moreover, fueling guidelines need to be tailored to energy expenditure to prevent a positive energy balance and weight gain. 

The main disciplines in para-cycling are road racing (up to 120 km), time trial (15 to 30 km), and the team relay (Table 1). For track cycling, shorter events such as 200 m sprint, 500 to 4000 m individual races, and scratch races (10 to 15 km) are scheduled. Para-cyclist would rely on endogenous CHO stores (e.g., liver and muscle glycogen) and exogenous supplementation (e.g., during road race) for energy production. No fluid or CHO intake is needed for shorter events (e.g., time trial, track races). A CHO loading (e.g., 10 to 12 g/kg/d for athletes with minimal disabilities) should be implemented in the 36 to 24 h preceding the road race to ensure optimal liver and muscle glycogen stores [45]. For the other disciplines, a carbohydrate-rich diet (7 to 10 g/kg/d) should be sufficient for CHO loading the day before the event. In addition, a light pre-event meal with a total amount of 1 to 4 g CHO/kg/d easily digestible CHO, low in fat and fiber, should be optimal for all other disciplines. Carbohydrate intake is extremely important to ensure a fast recovery and a glycogen refilling after the event. Therefore, the intake of 1 to 1.2 g CHO/kg/h for the next 4 h is recommended [45]. This is even more important during the World Cup or Paralympic Games, whereas athletes race two to three events over 3 days (e.g., handcycling: team relay, time trial, and road race). In professional able-bodied cyclists, low energy availability and poor CHO intake during and post-competition has been shown [54]. Thus, para-cycling athletes should apply optimized race nutrition and recovery plan, including refueling and rehydration. 

### 4.3. Protein Ingestion to Support Adaptation and Recovery

As for CHO intake, literature on protein intake and supplementation in Paralympic athletes is scarce, although it is well-studied in able-bodied athletes [5]. From athletes without disabilities, it seems obvious that protein intake is important regarding lean body mass, repair, remodeling, and adaptation to strength training [45]. Not only the amount of protein is important, but also the quality and timing. Animal compared to plant-based sources are known to have a better profile of essential amino acids and might be more beneficial when it comes to muscle protein synthesis and adaptation [55]. Whey protein, as an example, is very well studied and most effective in a dose of 20 to 25 g/dose or 0.3 to 0.4 g/kg/meal to stimulate muscle protein synthesis to a maximal extent consumed following a strength training or an intensive session [45,56]. No study on protein supplementation and its effect on muscle protein synthesis following a training session has been performed in Paralympic athletes [57]. Nevertheless, it seems reasonable to implement a post-session protein intake (20 to 25 g of high-quality protein or 0.3 to 0.4 g/kg/meal) to enhance recovery and adaptation in athletes [58,59]. Moreover, planning three to four protein servings over the whole day might further help to improve overall muscle protein synthesis and to minimize muscle protein breakdown [56]. Overall protein intake is recommended to be at least 1.2 g/kg/d [57] in athletes with SCI und and between 1.2 up to 2.2 g/kg/d in athletes with a minimal disability or able-bodied athletes [56]. Protein intake should be increased in case of weight loss and energy restriction. In case of constipation or kidney disease, athletes should be cautious with protein intake [57]. Athletes with SCI and amputation suffering from pressure ulcers or sores might also increase their daily protein intake to 2.0 g/kg/d [60]. Conclusively, planning of protein-rich sources and their implementation into training and racing schedule might be important to induce optimal training stimulus, recovery, and lean body mass in para-cycling athletes. Low caloric protein options for athletes with reduced energy needs (e.g., cottage or curd cheese, dried red meat) might be good fueling sources to achieve protein goals. 

### 4.4. Hydration

An hydrated state is a first step to optimizing performance in athletes [52]. Urine color seems to be a practical tool to check hydration status by the athletes through self-monitoring during the first urine in the morning. Only for athletes with visual impairment and those using urine collecting bags, urine color might not be optimal to rely on [52]. Thirst is a good indicator for the hydration status too. To ensure an appropriate hydration status, planning fluid intake would be beneficial. Furthermore, sufficient fluid ingestion can also prevent obstipation and urinary tract infections in athletes with SCI. Pritchett, Broad, Scaramella and Baumann [52] suggests consuming small salted snacks such as pretzels or fluids containing salt (20 to 50 mmol/L or 460 to 1150 mg/L) before exercise to help stimulate thirst and fluid retention. 

As there is little evidence for the effectiveness of sports drinks in individuals with SCI [61], this does not imply that CHO solutions do not help to increase performance in these athletes. The metabolic cost of upper body exercise is lower compared to whole-body exercise. Therefore, the use of sports drinks needs to be applied according to energy expenditure and individual sweating capacities to prevent weight gain and overdrinking [62]. Furthermore, gastric emptying might be reduced in individuals with SCI, increasing the risk of GI side effects [50]. No study has investigated sweat rate in handcycling athletes with SCI. Price and Campbell [63] have assessed sweat rate and fluid ingestion at 31 °C in various wheelchair athletes. They found a sweat rate of 0.3 L/h in individuals with tetraplegia and 0.7 L/h in athletes with high and low paraplegia. In a professional cyclist, sweat rate was 1.4 L/h during ambient (19 °C, 49%rh) and 1.7 L/h in hot (30 °C, 60%rh) conditions [64]. Dependent on the disability, the sweat rate will be reduced in most para-cycling athletes with the highest reduction in athletes with a tetraplegia [63]. This shows why individual drinking strategies are warranted. 

All para-cyclists, whether those with SCI, CP, visual impairment or amputees, should aim to prevent fluid loss to 2% of body weight loss [62]. It is recommended to use an individual approach to avoid overdrinking (e.g., athletes with low sweat rate), dehydration (e.g., high sweat rate), exercise-induced heat stress, maintain bladder and bowel routine and consider individual gastric emptying rate to avoid GI discomfort. 

### 4.5. Weight Loss and Energy Availability

Optimizing weight and body composition to perform well is also an aim in para-cycling, especially for endurance events. Often, athletes will consume less energy to restrict calories to induce weight loss [65]. This might lead to micronutrient deficiencies [19,47] and to reduced energy availability [5,21,66,67]. Egger and Flueck [21] showed a low energy availability in wheelchair athletes from various disciplines with a higher prevalence in female athletes. A survey in para-athletes showed menstrual dysfunction and reduced bone health in this population [65]. Cycling as a sport per se seems to be susceptible to subclinical eating disorders with a high prevalence in male cyclists [68]. A chronic low energy availability induces acute or long-term health impairments such as suppression of hormone production, compromised immunity, GI discomfort, menstrual dysfunction, impaired bone health, a reduction in training response and adaptation, and psychological issues (i.e., depression) [69]. Very little is known about energy availability and its definition or signs and symptoms in athletes with a disability. Of particular concern are athletes with SCI who often show signs of low bone mineral density and menstrual dysfunction due to their disability [65]. It seems reasonable to plan weight loss over a longer time to ensure a sufficient nutrient intake and stay healthy while inducing weight loss. Possibly, the amount of a negative energy balance needs to be adapted in the case of Paralympic athletes with already reduced energy needs. As suggested by several different authors [70], a periodized nutrition approach might help achieve this aim in athletes with a disability. It is suggested to plan the nutrition around the training schedule and to fuel the athletes with energy when needed to ensure optimal training conditions and adaptations as well as to enhance recovery [70]. 

### 4.6. Supplement Use

During the 2004 Paralympic Games, para-athletes declared a 64% use of supplements or medication [71]. Similar results were obtained by Flueck and Perret [72], where 63% and 43% of the athletes responded to use dietary supplements during training or competition phases, respectively. Supplement use was shown to be even higher (more than 85%) in able-bodied athletes from various different sports [73]. One might expect an increase in supplement use in Paralympic athletes due to sport professionalism. See Table 5.

As mentioned above, individuals with SCI are at risk for micronutrient deficiencies. Thus, the use of dietary supplements in athletes with SCI is not surprising to counteract deficiencies. Performance-enhancing supplements such as caffeine, creatine, bicarbonate, and sodium nitrate/beetroot juice have not been studied extensively in athletes with SCI (Table 5). Due to the physiological adaptations following an SCI, including a prolonged GI transition [51], more type I muscle fibers in the upper extremities [84], and the physiological differences between upper and lower body exercise, studies investigating the effectiveness of the supplements in this population are needed. As an example, a reduced epinephrine and norepinephrine concentration was shown in individuals with SCI at rest and during exercise [85]. Following caffeine ingestion, Flueck, Lienert [75] showed a difference in epinephrine and norepinephrine concentrations in able-bodied, para- and tetraplegic individuals. Furthermore, 6 mg per kg body mass of caffeine ingestion did not increase mean power output during a 3-min all-out test in all individuals even though power output was higher in the first 30 to 60 s in able-bodied and paraplegic except for tetraplegic individuals. This shows that we need to be cautious when recommending performance-enhancing supplements to athletes with SCI. In contrast, athletes with an amputation or a visual impairment are not expected to show a different mechanism of action as compared to able-bodied athletes. Therefore, these athletes should adhere to the IOC’s common guidelines for supplement use [86]. The other athletes (i.e., SCI, cerebral palsy) should choose an individual approach through guidance with a sports nutritionist or a dietician to make an evidence-informed decision. Secondary complications (i.e., kidney problems), GI issues, or a drug interaction should be considered to avoid harmfulness (e.g., creatine). 

### 4.7. Exercise in the Heat

The review by Griggs, Stephenson, Price and Goosey-Tolfrey [53] showed that para-cyclists are at the highest risk for heat-induced illness at the Paralympic Games in Tokyo. A number of different factors might contribute to this risk, including the environmental conditions, the characteristics of the sports itself (e.g., type, intensity, duration), the athlete’s fitness level, and the physical attributes (e.g., body composition). Clothing can further increase the risk for overheating due to a lower dissipation of heat through the skin and, as discussed above, the reduced ability to sweat in some individuals. 

Several different studies and review articles have shown that individuals with a SCI have a reduced capacity to lose heat due to a lack of sympathetic vasoconstriction and a limited ability to redistribute blood [53,87]. Especially athletes with a SCI have a reduced ability to cool their body through sweat loss. This is mainly dependent on their lesion level and the completeness of the lesion. Thus, athletes with tetraplegia are at a much higher risk of heat-related illnesses than those with paraplegia [87]. This was shown in a case study of a handcycling marathon [36], whereas rectal core temperature increased to 40 °C during the race in the athlete with tetraplegia. Similar to athletes with a SCI; also athletes with CP might experience these issues due to physiological and cognitive differences [6]. A pacing strategy might be altered, and therefore, with a faster start, the risk for exercise-induced heat illness increases in extreme environmental conditions [53]. Moreover, athletes with an amputation show a reduced capacity to dissipate heat due to a lower skin surface and ability to sweat [6]. Furthermore, they show a higher ability to elevate heat production at the transition from the prosthetics to the limb. This has not been confirmed yet in para-cyclists using prostheses during racing. Finally, athletes with a visual impairment need feedback on urine color, pacing, and hydration strategies during the race, especially in hot conditions [53]. 

To summarize, all athletes participating in para-cycling races are at risk for heat-related illness [53]. They should regularly monitor hydration status through urine color, urine specific gravity, or personal assistance (i.e., visually impaired). Furthermore, individual sweat loss should be checked in similar environmental conditions as the major competition is expected to happen (e.g., pre-and post-exercise body mass and drinking volume during the session). In athletes with reduced sweat capacity, overdrinking should be prevented, and cooling strategies might induce heat loss. Cooling vests and garments and pre- or post-exercise ice slurries might help decrease body core temperature. Furthermore, the use of menthol as a perceptual cooling strategy needs to be considered [88]. However, these athletes, especially individuals with SCI, might be more prone to GI side effects due to altered GI function. It is advised to test all interventions prior to competition and to train the GI system to minimize side effects. 

## 5. Practical Application

To ensure an evidence-based, impairment-specific nutritional counseling, the practical application is summarized in Table 6 regarding all discussed topics of this review. Energy demands should be estimated individually. It seems worthwhile to use indirect calorimetry to measure REE as no REE equation was developed for athletes with SCI or amputation [16]. Together with data from the training sessions and existing literature (Table 2) on energy expenditure during training [9], energy needs might be estimated in order to plan the nutritional intervention. A periodized nutrition approach [70] might further help to fuel for the specific training to enhance performance and optimize recovery without an excessive energy intake leading to weight gain. Before, during, and after high intensity or long endurance rides, athletes should make sure that they consume enough CHO in order to meet their requirements (Table 4). Furthermore, training their race fueling strategy might help decrease GI discomfort symptoms and train the GI system. Athletes should consume 4 to 5 servings of high-quality, protein-rich sources to support adaptation and recovery as well as to induce satiety (Table 4). To monitor body composition, the sum of skinfolds (without any equation) or the DXA scan might be applicable in para-cycling athletes. Hydration status might be checked and monitored using urine color and urine specific gravity [62]. The use of pre-and post-exercise body mass to calculate sweat rate and dehydration status during a training session. In all athletes but especially those with reduced sweat capacity (i.e., tetraplegia), cooling strategies (i.e., cooling vests and garments, ice slurries, and menthol) should be applied in hot conditions [53] to reduce the risk for exercise-induced heat illness. Especially during longer events (e.g., road race) and in extreme environmental conditions, cooling strategies should be implemented into pre/during/post nutrition strategy.

## 6. Limitations and Future Directions

This is the first review article summarizing the nutritional needs of para-cycling athletes. Scientific evidence in this population is scarce, and drawing any firm conclusion is difficult. The limited access to this population due to a lower number of athletes worldwide makes it difficult to develop population and sport-specific guidelines in terms of sports nutrition. Especially for upper body exercise and athletes with an impaired GI system (i.e., athletes with SCI), the optimal CHO intake during exercise, protein intake, and hydration need to be tailored individually. Furthermore, supplementation studies investigating the effectiveness of para-cycling are needed (e.g., long-term creatine supplementation, bicarbonate loading protocols to minimize GI issues, the effect of caffeine on endurance performance in SCI, etc.). 

## 7. Conclusions

Para-cycling involves athletes with different impairment types in endurance (e.g., road) and high-intensity exercise (e.g., track) involving upper or lower body muscles, so nutritional advice needs to be tailored for each individual and sport-specific situation. This review showed how complex and difficult it is to consider all physiological adaptations and demands while giving nutritional recommendations for each disability or racing category. Especially, the balance between energy expenditure and dietary intake and a sufficient micronutrient status (e.g., vitamin D, calcium, iron) seems to be important. Furthermore, internal (e.g., lesion level, sweat capacity, active muscle mass) and external factors (e.g., heat, humidity, altitude) must be considered when working with this special population. To summarize, CHO intake before, during, and after exercise and an adequate protein intake seem crucial to optimize performance, recovery, and lean body mass. Until studies on macronutrient intake have been carried out before, during, and after exercise, it is important to adhere to the general recommendations from able-bodied athletes and to adapt and tailor those individually. 

## Figures and Tables

**Table 1:** Classification and racing profile in para-cycling athletes.

**Road**				
**Classification**	**Impairment**	**Race Types**	**Yearly Training**	**Material**
C1–C5	Cerebral palsy, amputees, and other conditions who can ride a bike	Road race (men: 75–100 km; women: 60–75 km) Time trial (men: 25–30 km; women: 20–25 km)	300–800 h	Road bike
T1–T2	Cerebral palsy, neurological conditions or other athletes who are unable to ride a normal bike	Road race (men: 30–40 km; women: 30 km) Time trial (men: 15–20 km; women: 15 km)	350–450 h	Tricycle
B	Visually impaired	Road race (men: 120 km; women: 100 km) Time Trial (men: 35 km; women: 30 km)	700–800 h	Tandem
H1–H5	Impairments affecting both legs or a combination of the upper and lower limbs (amputees, paraplegia, tetraplegia)	Road race (men: 60–80 km; women: 50–80 km) Time trial (men: 20–30 km; women: 20–30 km) Team relay	250–550 h	Handcycle
**Track**				
**Classification**	**Impairment**	**Race Types**	**Yearly Training**	**Material**
C1–C5	Cerebral palsy, amputees, and other conditions who can ride a bike	1000 (men)/500 m (women) Individual pursuit (men: 3000–4000 m; women: 3000 m Team sprint Scratch Race (men: 15 km; women 10 km)	300–800 h	Track bike
B	Visually impaired	1000 m (men & women) Individual pursuit (men: 4000 m; women: 3000 m) Sprint 200 m	700–800 h	Tandem

Data extracted from Cycling Canada [10] and Union Cycliste Internationale [11].

**Table 2:** Energy expenditure during handcycling and arm cranking in individuals with a spinal cord injury.

**Handcycling**				
**Author**	**Group**	**Energy Expenditure**	**CHO (g/min)**	**Fat (g/min)**
Abel, Schneider, Platen and Strüder [36]	Handbike marathon race (case study)	7.7 kcal/min	N/A	N/A
Abel, Kroner, Rojas Vega, Peters, Klose and Platen [17]	Handbiking at 2 mmol/L LT and at 4 mmol/L LT	2 mmol/L LT: 6.5 kcal/min 4 mmol/L LT: 8.8 kcal/min	N/A	N/A
**Arm Cranking**				
**Author**	**Group**	**Energy expenditure**	**CHO (g/min)**	**Fat (g/min)**
Price and Campbell [37]	SCI & AB, 80% HRpeak	SCI: 6.5 kcal/min AB: 9.1 kcal/min	SCI: 1.45 AB: 1.90	SCI: 0.19 AB: 0.13
Price and Campbell [38]	SCI, 60% VO2max	6.6 kcal/min	0.95	0.29
Knechtle and Kopfli [39]	SCI (arm crank) & AB (cycling) 55/65/75% VO2peak	SCI: 6.5/7.5/9.0 kcal/min AB: 12.5/15.0/17.5 kcal/min	SCI: 1.1/1.4 /1.6 AB: 1.8/2.2/2.6	SCI: 0.22/0.22/0.25 AB: 0.55/0.6/0.7

Note: LT = lactate threshold; AB = able-bodied, SCI = spinal cord injury, CHO = carbohydrate oxidation, Fat = fat oxidation, N/A = not applicable.

**Table 3:** Example of a nutrition plan for a 50 kg female para-cycling athlete (H3 category) with a spinal cord injury focused on CHO and protein intake.

**Breakfast (6.30 a.m.)**	2 slices of wholegrain bread, 2 eggs, butter and jam, green tea	50 g CHO, 15 g protein
**Training Session (8.30 a.m.)**	90 min gym session (strength training) Water during the session	
**Post-Training Snack (10 a.m.)**	Whey protein powder with water	20 g protein
**Lunch (12.15 p.m.)**	Small bowl of rice with vegetables and sliced chicken (60 g)	50 g CHO, 20 g protein
**Training Session (2 p.m.)**	120 min moderate to high intensity endurance session 1 L sports drink	90 g CHO (45 g/h), 1 L water (0.5 L/h)
**Post-Training Snack (4.15 p.m.)**	Self-made smoothie with milk, curd cheese or greek yogurt, fruits and oats	50 g CHO, 15 g protein
**Dinner (6.30 p.m.)**	1 piece of salmon, potatoes cooked in salty water, vegetables, fruit as a dessert	60 g CHO, 20 g protein
**Daily Consumption**		300 g CHO = 6.0 g CHO/kg/d 80 g protein = 1.6 g/kg/d

Note: CHO = carbohydrate, resting energy expenditure = 1450 kcal/d, exercise energy expenditure predicted = 800 kcal.

**Table 4:** Nutritional and thermoregulatory considerations for para-cycling athletes.

**Issue**	**Description**	**Recommendation**
General Fueling	Low-intensity training, low volume	3 to 5 g CHO/kg/day
	Moderate intensity training (e.g., 1 h/d moderate to high intensity)	5 to 7 g CHO/kg/day
	High-volume and/or intensity (e.g., 1–3 h/d)	6 to 10 g CHO/kg/day
	Very high-volume, high-intensity training load (e.g., >4–5 h/d)	8 to 12 g CHO/kg/day
	Protein	1.2 to 1.8 g/kg/day
Pre Session	>60 min before the session	1 to 4 g CHO/kg (1–4 h before)
During Session	<45 min	No CHO or fluid needed
	45 to 75 min	Water or small amounts of CHO including CHO mouth wash (CHO rinse for 60 s)
	60 to 150 min	30 to 60 g CHO/h; water amount based on individual sweat rate
	>150 min	Up to 90 g CHO/h, a combination of glucose and fructose
Post Session	CHO reloading	1 to 1.2 g CHO/kg/h, for the first 4 h
	Rehydration	150% of fluid losses consumed over the next few hours, using salty snacks in the heat or with “salty sweating” athletes
	Protein (i.e., repair, muscle protein synthesis)	20 to 25 g of high-quality protein (or 0.3 to 0.5 g/kg)
Hydration	Monitor hydration status via first morning urine (e.g., urine color, USG) or via thirst	Pale urine color in the morning, USG <1.020
	Monitor pre/post-session body weight (cave: wear minimal clothing) to estimate fluid loss and sweat rate	Body mass loss <2% Avoid overdrinking (e.g., no weight gain)
Heat	Pre-cooling	Ice slurry, ice towel, cold water immersion, ice vest (60 to 20min before the exercise)
	Per-Cooling	Ice vests, ice towel, artificial sweating (e.g., water spray), menthol, cooling garments
	Sweat capacity	Sweat capacity impaired in athletes with SCI (i.e., tetraplegia) and amputees → individual strategies to pre-or per-cooling needed
	Hyperhydration	Using a salt or glycerol loading protocol to increase plasma volume (e.g., high sweat rates, salty sweater, hot conditions)

Adapted from [5,6,45,52,53] Note: CHO = carbohydrate, USG = urine specific gravity.

**Table 5:** Dietary supplement application in wheelchair athletes adapted from Broad et al. (2019) [6].

Supplement	Author	Dosage	Protocol	Outcome
Caffeine	Flueck, Mettler and Perret [74]	6 mg per kg body mass caffeine 0.5 g per kg body mass sodium citrate 120 min (sodium citrate) and 60 min (caffeine) before a test	9 wheelchair athletes (6 male, 3 female) 4 × 1500 m time trial on wheelchair training roller (placebo, caffeine, sodium citrate, sodium citrate + caffeine)	No significant difference in performance
	Flueck, Lienert, Schaufelberger, Krebs and Perret [75]	6 mg per kg body mass caffeine, 60 min before the test	17 AB, 10 PA, 7 tetraplegic (all recreationally active) 3 min all-out test at arm crank ergometer	Significant higher power output first 30 and 60 s (in AB and PA), average power output over 3 min not significantly altered
	Graham-Paulson, Perret, Watson and Goosey-Tolfrey [76]	4 mg per kg body mass caffeine, 70 min before the test	12 wheelchair rugby players 4 × 4 min plus 3 × 3× 20 m sprint	Significantly faster sprint performance
	Graham-Paulson, Perret and Goosey-Tolfrey [77]	2, 4 and 6 mg per kg body mass caffeine Vs. placebo	Case study: 1 para-triathlete 20 km time trial	TT performance increased by 2, 1.7, and 2.5% compared to the placebo
Vitamin D	Flueck, Schlaepfer and Perret [78]	6000 IU Vitamin D3 over 12 weeks followed by 12 weeks of placebo ingestion	20 indoor wheelchair athletes (basketball and rugby); Wingate test Isometric strength test	Significantly increased vitamin D status; no significant difference in Wingate performance, a significant increase in isometric strength in the non-dominant arm
	Pritchett, Pritchett Stark, Broad and LaCroix [79]	<50 nmol/L: 50,000 IU/week for 8 weeks 50–75 nmol/L: 35,000 IU/week for 4 weeks Maintenance 15,000 IU/week >75 nmol/L: 15,000 IU/week	34 wheelchair athletes Handgrip strength 20 m sprint performance	91% of the athletes showed a sufficient vitamin D status, a significant increase in handgrip strength No change in sprint performance
Beetroot juice	Flueck, Gallo, Moelijker, Bogdanov, Bogdanova and Perret [80]	6 mmol beetroot juice or sodium nitrate or water (placebo)	8 handcyclists (category H2 to H4) 10 km time trial	Significant increase in plasma nitrate and nitrite concentrations, no significant effect on time to complete the trial
Creatine monohydrate	Perret, Mueller and Knecht [81]	4 × 5 g/day for 6 days	6 wheelchair athletes (4 male, 2 female) 800 m time trial	No significant differences in performance, lactate concentration, and heart rate
	Jacobs, Mahoney, Cohn, Sheradsky and Green [82]	4 × 5 g/day for 7 days	16 untrained patients with a chronic tetraplegia VO_2peak_ test at the arm-crank ergometer	Significant increase in VO_2peak_ (+17.4%) and peak power (+6.7%)
Carbohydrate	Spendiff and Campbell [61]	600 mL of a low (4%) and a high (11%) carbohydrate drink, 20 min before exercise	8 male wheelchair athletes; 1 h exercise at 65% VO_2peak_ followed by 20 min time trial	The tendency for a greater performance
Fish-oil	Marques et al. [83]	3 g fish oil (1500 mg DHA, 300 mg EPA) per day for 30 days	8 male wheelchair basketball players; inflammation markers before and after exercise	Reduced markers of muscle damage, inflammatory disturbances, and neutrophil death

Note: AB = able-bodied, PA = paraplegic participant.

**Table 6:** Practical application of nutritional assessments and interventions in para-cycling athletes with different impairments.

Issue	Description	Recommendation
Energy needs	Evaluation of energy needs based on the physiological adaptations because of the disability	Measurement of resting energy expenditure by indirect calorimetry Sport-specific measurement of exercise energy expenditure by indirect calorimetry or estimation of metabolic cost based on available scientific literature Plan energy intake based on a training schedule to ensure optimal performance and adaptation
Body composition	Monitoring of body composition to track balance between energy needs and dietary intake	Use DXA measurement or sum of skinfolds to monitor No specific equation for athletes with a disability (e.g., to estimate fat-free mass based on skinfold)
Micronutrient status	Monitoring of micronutrient intake and status as athletes with a disability seems to be at a higher risk for micronutrient deficiencies	Monitor micronutrient intake through a food diary Monitor vitamin D (25[OH]D) and ferritin levels regularly
Supplement use	Education of the athletes concerning the list of prohibited substances Impairment-specific use of supplements Drug-supplement interactions	Use of supplements based on given scientific evidence Test the supplement during a training session (i.e., GI issues, mechanisms of action, objective and subjective measures, performance) Ask for the medication list of the athlete, review the existing literature on drug-supplement interactions Review patient history (or consult a physician) to ensure safe application
Secondary complications	Prevention of secondary complications such as urinary tract infections, GI issues, respiratory tract infections, and pressure ulcers (most common secondary complications in Paralympic athletes)	Educate the athletes on how to prevent secondary complications Regular monitoring of skin surface Monitor hydration status (as recommended in Table 4) Use of prevention measures (e.g., pro-and prebiotics, cranberry juice) Monitoring of illness and injury by using a weekly screening questionnaire before a major competition to ensure early detection of complications

DXA = Dual-Energy X-ray absorptiometry, GI = gastrointestinal.

## Data Availability

Not applicable.
